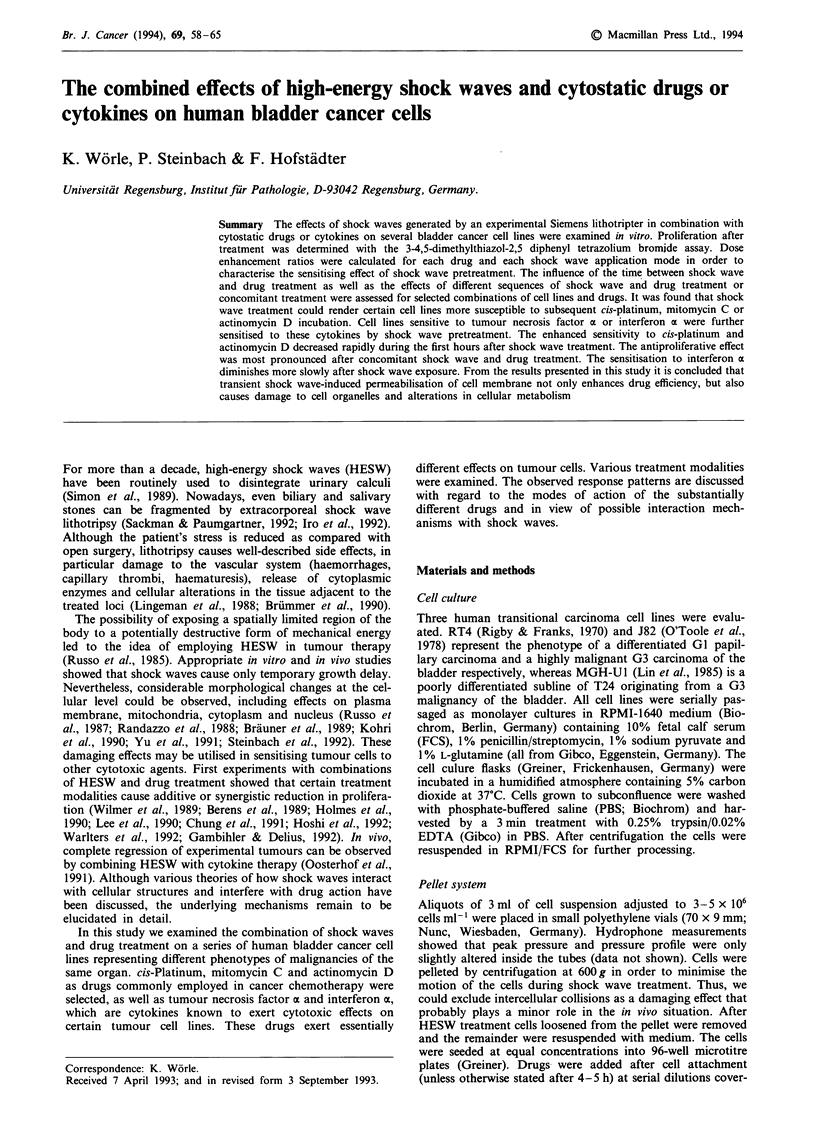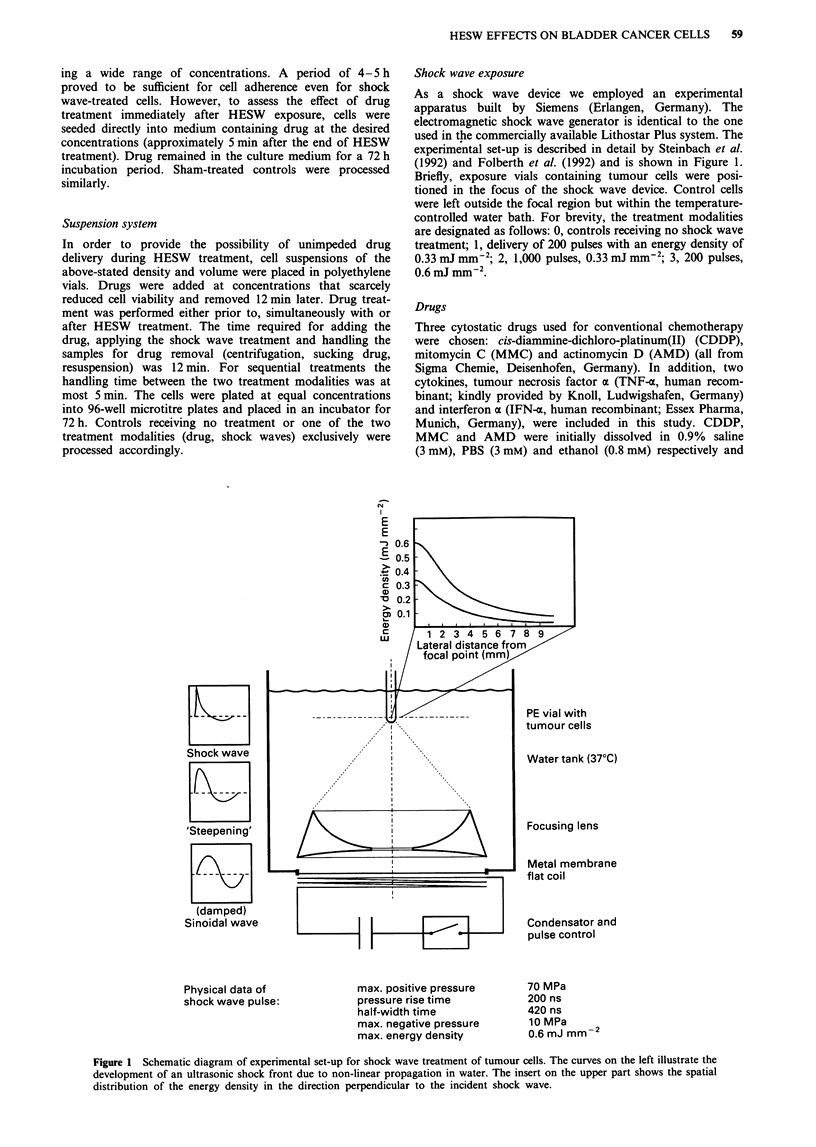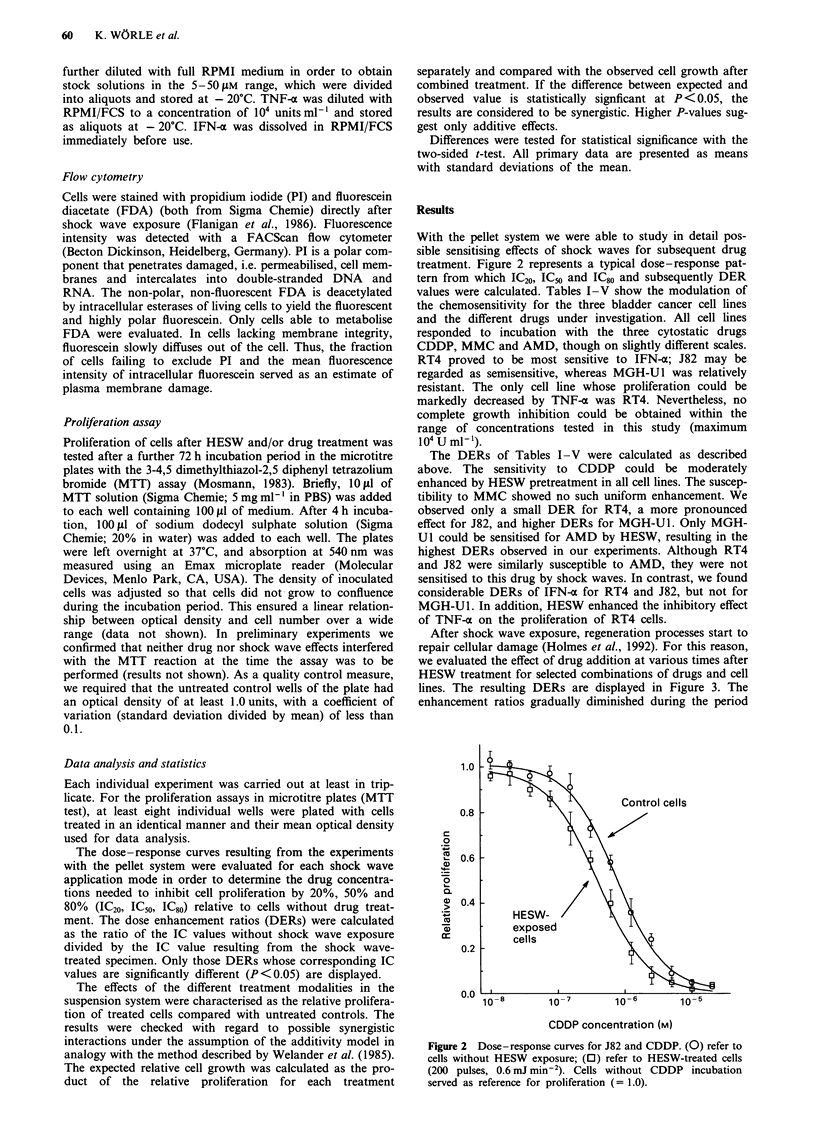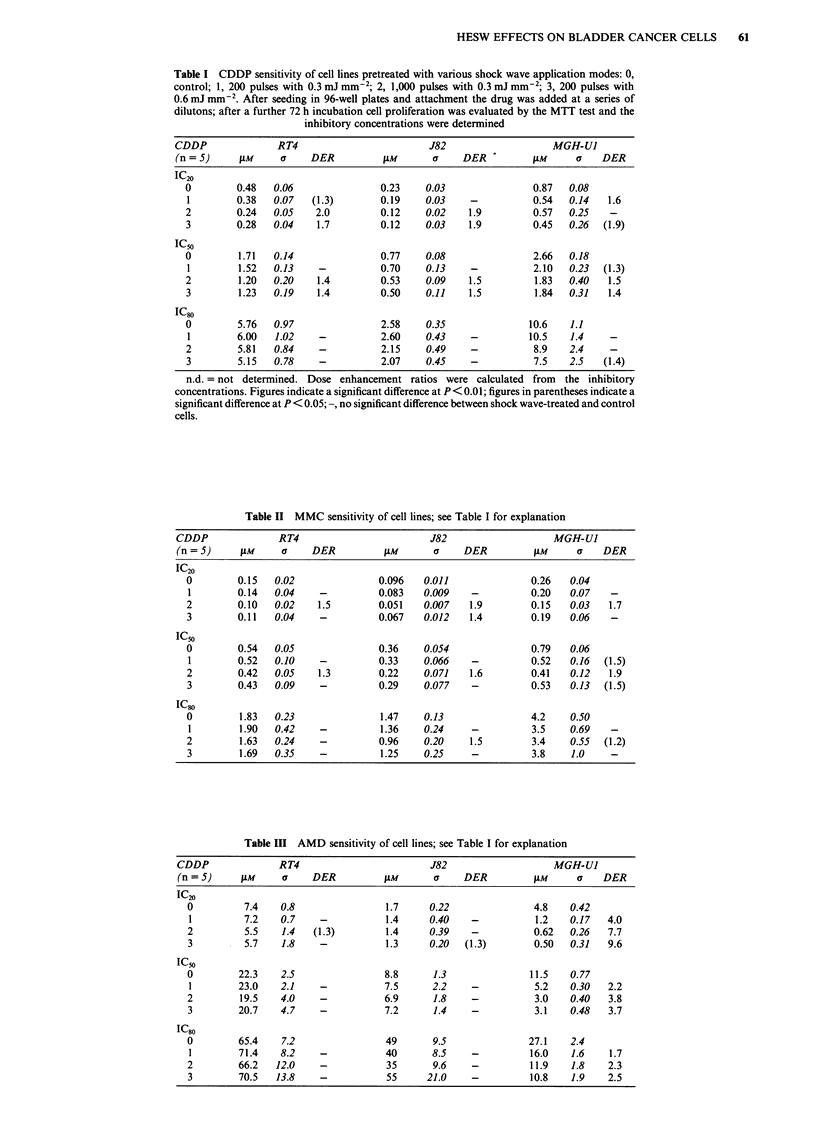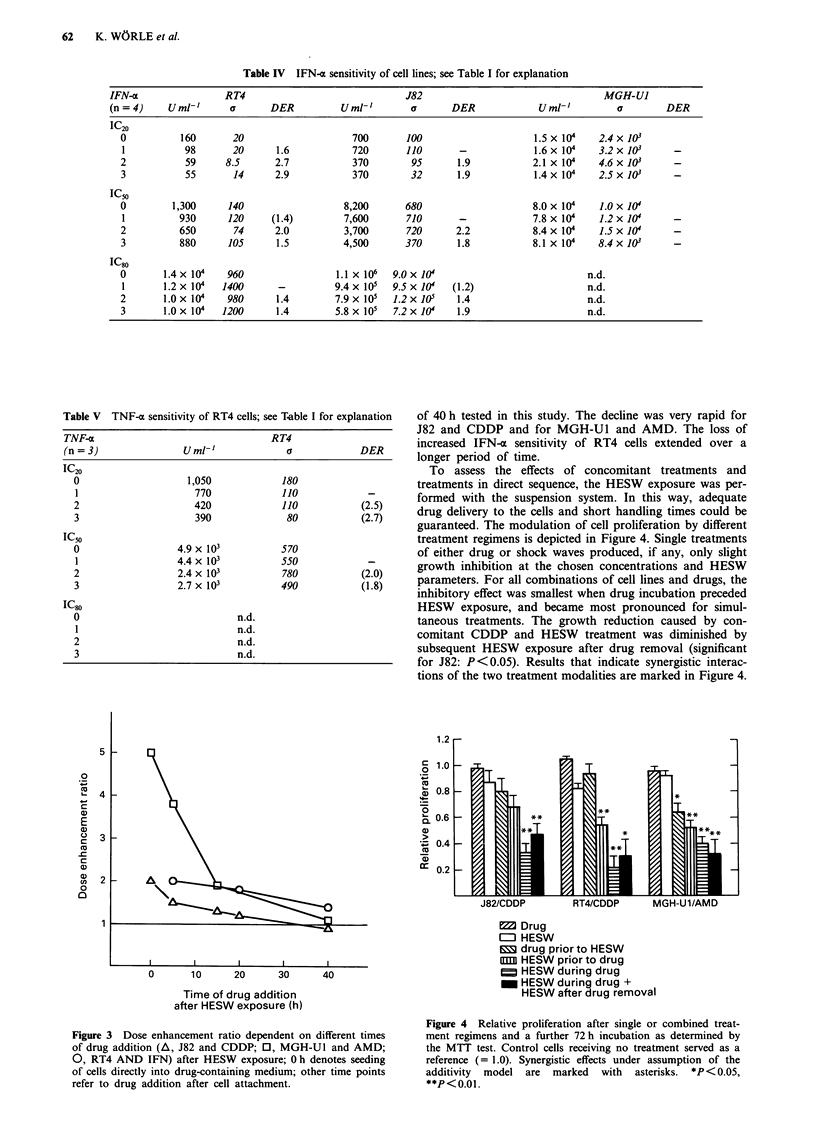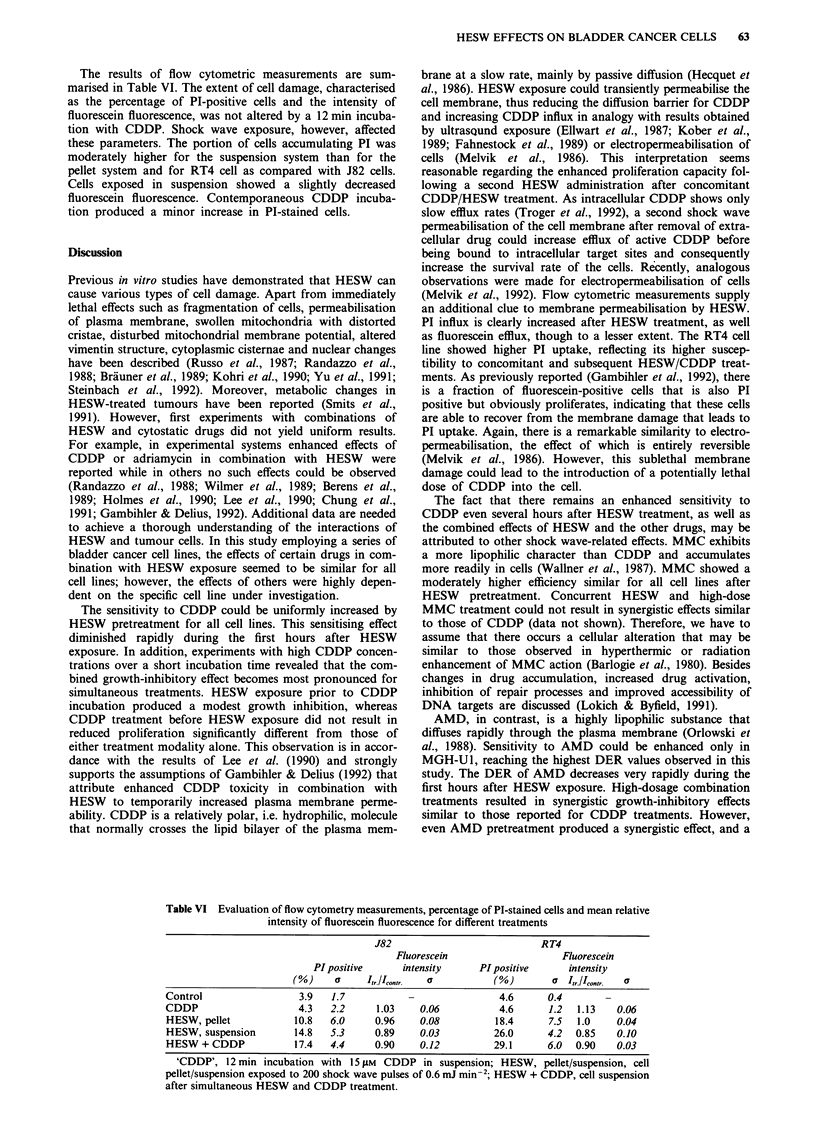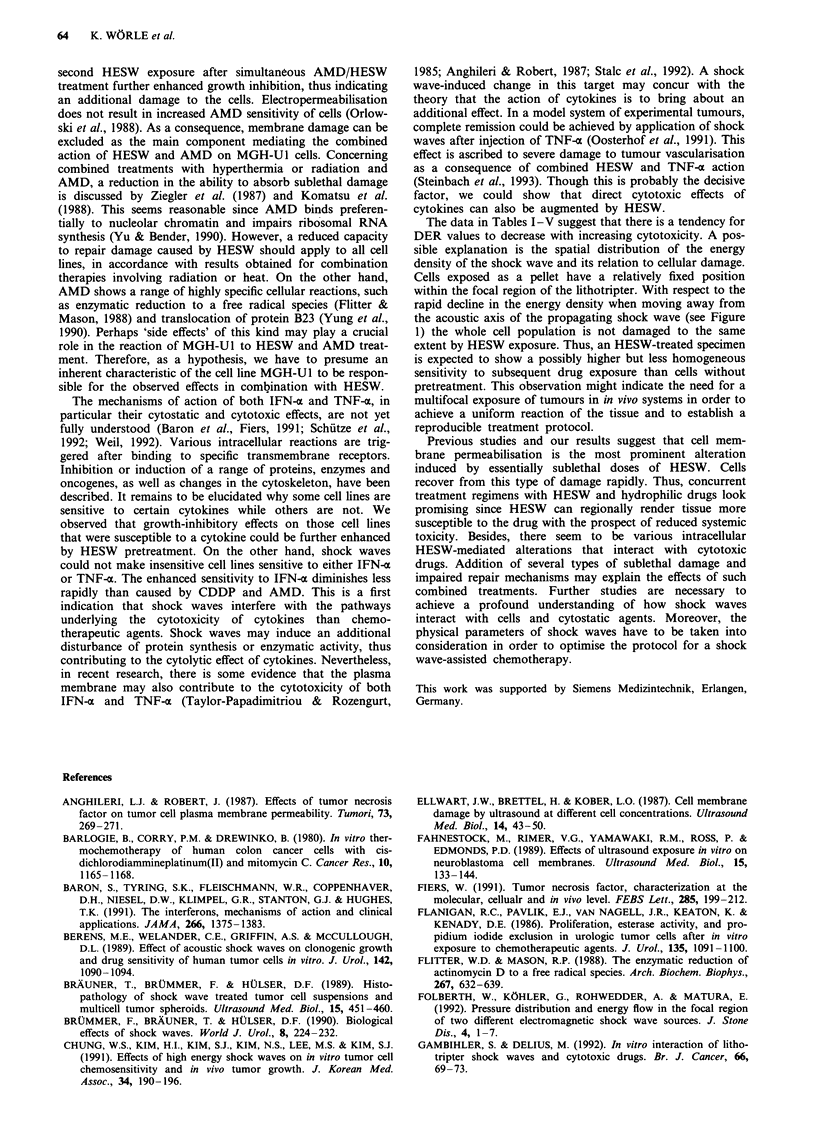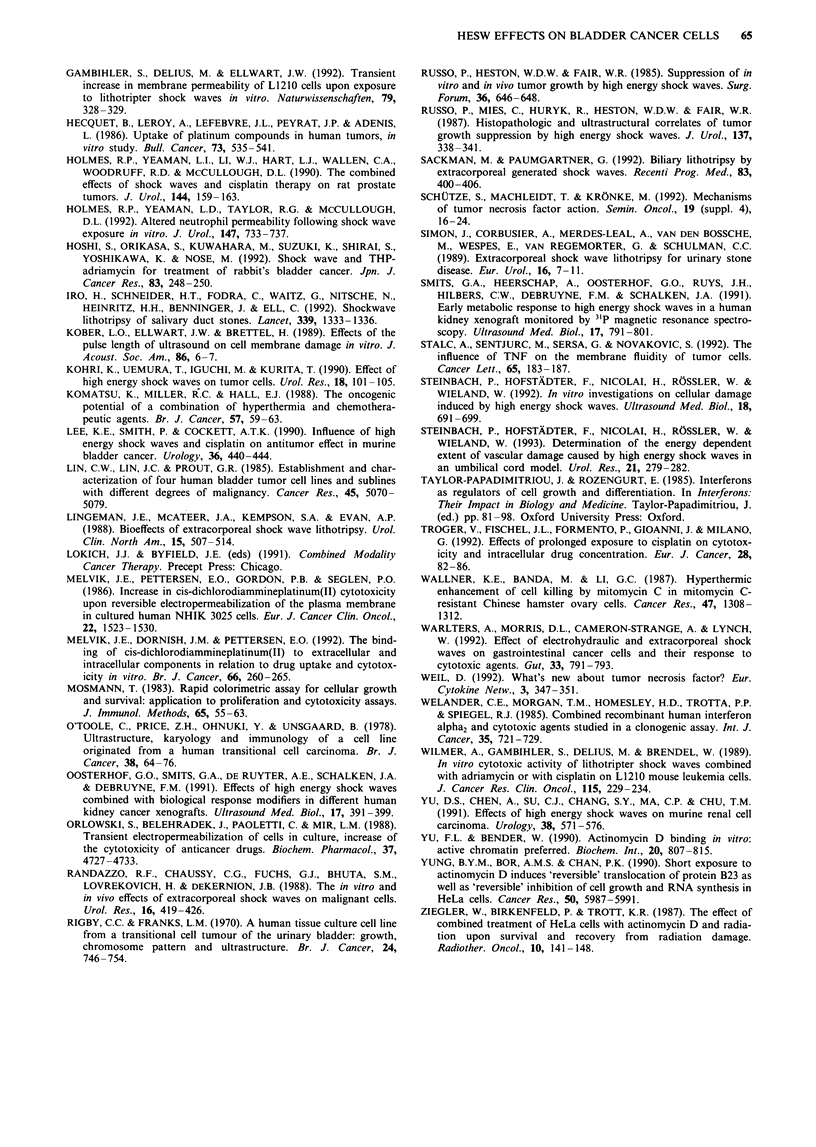# The combined effects of high-energy shock waves and cytostatic drugs or cytokines on human bladder cancer cells.

**DOI:** 10.1038/bjc.1994.9

**Published:** 1994-01

**Authors:** K. Wörle, P. Steinbach, F. Hofstädter

**Affiliations:** Universität Regensburg, Institut für Pathologie, Germany.

## Abstract

The effects of shock waves generated by an experimental Siemens lithotripter in combination with cytostatic drugs or cytokines on several bladder cancer cell lines were examined in vitro. Proliferation after treatment was determined with the 3-4,5-dimethylthiazol-2,5 diphenyl tetrazolium bromide assay. Dose enhancement ratios were calculated for each drug and each shock wave application mode in order to characterise the sensitising effect of shock wave pretreatment. The influence of the time between shock wave and drug treatment as well as the effects of different sequences of shock wave and drug treatment or concomitant treatment were assessed for selected combinations of cell lines and drugs. It was found that shock wave treatment could render certain cell lines more susceptible to subsequent cis-platinum, mitomycin C or actinomycin D incubation. Cell lines sensitive to tumour necrosis factor alpha or interferon alpha were further sensitised to these cytokines by shock wave pretreatment. The enhanced sensitivity to cis-platinum and actinomycin D decreased rapidly during the first hours after shock wave treatment. The antiproliferative effect was most pronounced after concomitant shock wave and drug treatment. The sensitisation to interferon alpha diminishes more slowly after shock wave exposure. From the results presented in this study it is concluded that transient shock wave-induced permeabilisation of cell membrane not only enhances drug efficiency, but also causes damage to cell organelles and alterations in cellular metabolism.


*Br. J. Cancer* (1994), **69**, 58–65 © Macmillan Press Ltd., 1994

### The combined effects of high-energy shock waves and cytostatic drugs or cytokines on human bladder cancer cells

K. Wörle, P. Steinbach & F. Hofstädter

*Universität Regensburg, Institut für Pathologie, D-93042 Regensburg, Germany.*

**Summary** The effects of shock waves generated by an experimental Siemens lithotripter in combination with cytostatic drugs or cytokines on several bladder cancer cell lines were examined *in vitro*. Proliferation after treatment was determined with the 3-4,5-dimethylthiazol-2,5 diphenyl tetrazolium bromide assay. Dose enhancement ratios were calculated for each drug and each shock wave application mode in order to characterise the sensitising effect of shock wave pretreatment. The influence of the time between shock wave and drug treatment as well as the effects of different sequences of shock wave and drug treatment or concomitant treatment were assessed for selected combinations of cell lines and drugs. It was found that shock wave treatment could render certain cell lines more susceptible to subsequent *cis*-platinum, mitomycin C or actinomycin D incubation. Cell lines sensitive to tumour necrosis factor α or interferon α were further sensitised to these cytokines by shock wave pretreatment. The enhanced sensitivity to *cis*-platinum and actinomycin D decreased rapidly during the first hours after shock wave treatment. The antiproliferative effect was most pronounced after concomitant shock wave and drug treatment. The sensitisation to interferon α diminishes more slowly after shock wave exposure. From the results presented in this study it is concluded that transient shock wave-induced permeabilisation of cell membrane not only enhances drug efficiency, but also causes damage to cell organelles and alterations in cellular metabolism

For more than a decade, high-energy shock waves (HESW) have been routinely used to disintegrate urinary calculi (Simon *et al.*, 1989). Nowadays, even biliary and salivary stones can be fragmented by extracorporeal shock wave lithotripsy (Sackman & Paumgartner, 1992; Iro *et al.*, 1992). Although the patient's stress is reduced as compared with open surgery, lithotripsy causes well-described side effects, in particular damage to the vascular system (haemorrhages, capillary thrombi, haematuresis), release of cytoplasmic enzymes and cellular alterations in the tissue adjacent to the treated loci (Lingeman *et al.*, 1988; Brümmer *et al.*, 1990).

The possibility of exposing a spatially limited region of the body to a potentially destructive form of mechanical energy led to the idea of employing HESW in tumour therapy (Russo *et al.*, 1985). Appropriate *in vitro* and *in vivo* studies showed that shock waves cause only temporary growth delay. Nevertheless, considerable morphological changes at the cellular level could be observed, including effects on plasma membrane, mitochondria, cytoplasm and nucleus (Russo *et al.*, 1987; Randazzo *et al.*, 1988; Bräuner *et al.*, 1989; Kohri *et al.*, 1990; Yu *et al.*, 1991; Steinbach *et al.*, 1992). These damaging effects may be utilised in sensitising tumour cells to other cytotoxic agents. First experiments with combinations of HESW and drug treatment showed that certain treatment modalities cause additive or synergistic reduction in proliferation (Wilmer *et al.*, 1989; Berens *et al.*, 1989; Holmes *et al.*, 1990; Lee *et al.*, 1990; Chung *et al.*, 1991; Hoshi *et al.*, 1992; Warlters *et al.*, 1992; Gambihler & Delius, 1992). *In vivo*, complete regression of experimental tumours can be observed by combining HESW with cytokine therapy (Oosterhof *et al.*, 1991). Although various theories of how shock waves interact with cellular structures and interfere with drug action have been discussed, the underlying mechanisms remain to be elucidated in detail.

In this study we examined the combination of shock waves and drug treatment on a series of human bladder cancer cell lines representing different phenotypes of malignancies of the same organ. *cis*-Platinum, mitomycin C and actinomycin D as drugs commonly employed in cancer chemotherapy were selected, as well as tumour necrosis factor α and interferon α, which are cytokines known to exert cytotoxic effects on certain tumour cell lines. These drugs exert essentially different effects on tumour cells. Various treatment modalities were examined. The observed response patterns are discussed with regard to the modes of action of the substantially different drugs and in view of possible interaction mechanisms with shock waves.

### Materials and methods

### *Cell culture*

Three human transitional carcinoma cell lines were evaluated. RT4 (Rigby & Franks, 1970) and J82 (O'Toole *et al.*, 1978) represent the phenotype of a differentiated G1 papillary carcinoma and a highly malignant G3 carcinoma of the bladder respectively, whereas MGH-U1 (Lin *et al.*, 1985) is a poorly differentiated subline of T24 originating from a G3 malignancy of the bladder. All cell lines were serially passaged as monolayer cultures in RPMI-1640 medium (Biochrom, Berlin, Germany) containing 10% fetal calf serum (FCS), 1% penicillin/streptomycin, 1% sodium pyruvate and 1% L-glutamine (all from Gibco, Eggenstein, Germany). The cell culure flasks (Greiner, Frickenhausen, Germany) were incubated in a humidified atmosphere containing 5% carbon dioxide at 37°C. Cells grown to subconfluence were washed with phosphate-buffered saline (PBS; Biochrom) and harvested by a 3 min treatment with 0.25% trypsin/0.02% EDTA (Gibco) in PBS. After centrifugation the cells were resuspended in RPMI/FCS for further processing.

### *Pellet system*

Aliquots of 3 ml of cell suspension adjusted to $3-5 \times 10^6$ cells $ml^{-1}$ were placed in small polyethylene vials (70 × 9 mm; Nunc, Wiesbaden, Germany). Hydrophone measurements showed that peak pressure and pressure profile were only slightly altered inside the tubes (data not shown). Cells were pelleted by centrifugation at 600 *g* in order to minimise the motion of the cells during shock wave treatment. Thus, we could exclude intercellular collisions as a damaging effect that probably plays a minor role in the *in vivo* situation. After HESW treatment cells loosened from the pellet were removed and the remainder were resuspended with medium. The cells were seeded at equal concentrations into 96-well microtitre plates (Greiner). Drugs were added after cell attachment (unless otherwise stated after 4–5 h) at serial dilutions cover-

Correspondence: K. Wörle.
Received 7 April 1993; and in revised form 3 September 1993.

ing a wide range of concentrations. A period of 4–5 h proved to be sufficient for cell adherence even for shock wave-treated cells. However, to assess the effect of drug treatment immediately after HESW exposure, cells were seeded directly into medium containing drug at the desired concentrations (approximately 5 min after the end of HESW treatment). Drug remained in the culture medium for a 72 h incubation period. Sham-treated controls were processed similarly.

*Suspension system*

In order to provide the possibility of unimpeded drug delivery during HESW treatment, cell suspensions of the above-stated density and volume were placed in polyethylene vials. Drugs were added at concentrations that scarcely reduced cell viability and removed 12 min later. Drug treatment was performed either prior to, simultaneously with or after HESW treatment. The time required for adding the drug, applying the shock wave treatment and handling the samples for drug removal (centrifugation, sucking drug, resuspension) was 12 min. For sequential treatments the handling time between the two treatment modalities was at most 5 min. The cells were plated at equal concentrations into 96-well microtitre plates and placed in an incubator for 72 h. Controls receiving no treatment or one of the two treatment modalities (drug, shock waves) exclusively were processed accordingly.

*Shock wave exposure*

As a shock wave device we employed an experimental apparatus built by Siemens (Erlangen, Germany). The electromagnetic shock wave generator is identical to the one used in the commercially available Lithostar Plus system. The experimental set-up is described in detail by Steinbach *et al.* (1992) and Folberth *et al.* (1992) and is shown in Figure 1. Briefly, exposure vials containing tumour cells were positioned in the focus of the shock wave device. Control cells were left outside the focal region but within the temperature-controlled water bath. For brevity, the treatment modalities are designated as follows: 0, controls receiving no shock wave treatment; 1, delivery of 200 pulses with an energy density of 0.33 mJ $mm^{-2}$; 2, 1,000 pulses, 0.33 mJ $mm^{-2}$; 3, 200 pulses, 0.6 mJ $mm^{-2}$.

*Drugs*

Three cytostatic drugs used for conventional chemotherapy were chosen: *cis*-diammine-dichloro-platinum(II) (CDDP), mitomycin C (MMC) and actinomycin D (AMD) (all from Sigma Chemie, Deisenhofen, Germany). In addition, two cytokines, tumour necrosis factor α (TNF-α, human recombinant; kindly provided by Knoll, Ludwigshafen, Germany) and interferon α (IFN-α, human recombinant; Essex Pharma, Munich, Germany), were included in this study. CDDP, MMC and AMD were initially dissolved in 0.9% saline (3 mM), PBS (3 mM) and ethanol (0.8 mM) respectively and

**Figure 1** Schematic diagram of experimental set-up for shock wave treatment of tumour cells. The curves on the left illustrate the development of an ultrasonic shock front due to non-linear propagation in water. The insert on the upper part shows the spatial distribution of the energy density in the direction perpendicular to the incident shock wave.

further diluted with full RPMI medium in order to obtain stock solutions in the 5–50 μM range, which were divided into aliquots and stored at − 20°C. TNF-α was diluted with RPMI/FCS to a concentration of $10^4$ units $ml^{-1}$ and stored as aliquots at − 20°C. IFN-α was dissolved in RPMI/FCS immediately before use.

*Flow cytometry*

Cells were stained with propidium iodide (PI) and fluorescein diacetate (FDA) (both from Sigma Chemie) directly after shock wave exposure (Flanigan *et al.*, 1986). Fluorescence intensity was detected with a FACScan flow cytometer (Becton Dickinson, Heidelberg, Germany). PI is a polar component that penetrates damaged, i.e. permeabilised, cell membranes and intercalates into double-stranded DNA and RNA. The non-polar, non-fluorescent FDA is deacetylated by intracellular esterases of living cells to yield the fluorescent and highly polar fluorescein. Only cells able to metabolise FDA were evaluated. In cells lacking membrane integrity, fluorescein slowly diffuses out of the cell. Thus, the fraction of cells failing to exclude PI and the mean fluorescence intensity of intracellular fluorescein served as an estimate of plasma membrane damage.

*Proliferation assay*

Proliferation of cells after HESW and/or drug treatment was tested after a further 72 h incubation period in the microtitre plates with the 3-4,5 dimethylthiazol-2,5 diphenyl tetrazolium bromide (MTT) assay (Mosmann, 1983). Briefly, 10 μl of MTT solution (Sigma Chemie; 5 mg $ml^{-1}$ in PBS) was added to each well containing 100 μl of medium. After 4 h incubation, 100 μl of sodium dodecyl sulphate solution (Sigma Chemie; 20% in water) was added to each well. The plates were left overnight at 37°C, and absorption at 540 nm was measured using an Emax microplate reader (Molecular Devices, Menlo Park, CA, USA). The density of inoculated cells was adjusted so that cells did not grow to confluence during the incubation period. This ensured a linear relationship between optical density and cell number over a wide range (data not shown). In preliminary experiments we confirmed that neither drug nor shock wave effects interfered with the MTT reaction at the time the assay was to be performed (results not shown). As a quality control measure, we required that the untreated control wells of the plate had an optical density of at least 1.0 units, with a coefficient of variation (standard deviation divided by mean) of less than 0.1.

*Data analysis and statistics*

Each individual experiment was carried out at least in triplicate. For the proliferation assays in microtitre plates (MTT test), at least eight individual wells were plated with cells treated in an identical manner and their mean optical density used for data analysis.

The dose–response curves resulting from the experiments with the pellet system were evaluated for each shock wave application mode in order to determine the drug concentrations needed to inhibit cell proliferation by 20%, 50% and 80% ($IC_{20}$, $IC_{50}$, $IC_{80}$) relative to cells without drug treatment. The dose enhancement ratios (DERs) were calculated as the ratio of the IC values without shock wave exposure divided by the IC value resulting from the shock wave-treated specimen. Only those DERs whose corresponding IC values are significantly different ($P<0.05$) are displayed.

The effects of the different treatment modalities in the suspension system were characterised as the relative proliferation of treated cells compared with untreated controls. The results were checked with regard to possible synergistic interactions under the assumption of the additivity model in analogy with the method described by Welander *et al.* (1985). The expected relative cell growth was calculated as the product of the relative proliferation for each treatment separately and compared with the observed cell growth after combined treatment. If the difference between expected and observed value is statistically signficant at $P<0.05$, the results are considered to be synergistic. Higher *P*-values suggest only additive effects.

Differences were tested for statistical significance with the two-sided *t*-test. All primary data are presented as means with standard deviations of the mean.

### Results

With the pellet system we were able to study in detail possible sensitising effects of shock waves for subsequent drug treatment. Figure 2 represents a typical dose–response pattern from which $IC_{20}$, $IC_{50}$ and $IC_{80}$ and subsequently DER values were calculated. Tables I–V show the modulation of the chemosensitivity for the three bladder cancer cell lines and the different drugs under investigation. All cell lines responded to incubation with the three cytostatic drugs CDDP, MMC and AMD, though on slightly different scales. RT4 proved to be most sensitive to IFN-α; J82 may be regarded as semisensitive, whereas MGH-U1 was relatively resistant. The only cell line whose proliferation could be markedly decreased by TNF-α was RT4. Nevertheless, no complete growth inhibition could be obtained within the range of concentrations tested in this study (maximum $10^4$ U $ml^{-1}$).

The DERs of Tables I–V were calculated as described above. The sensitivity to CDDP could be moderately enhanced by HESW pretreatment in all cell lines. The susceptibility to MMC showed no such uniform enhancement. We observed only a small DER for RT4, a more pronounced effect for J82, and higher DERs for MGH-U1. Only MGH-U1 could be sensitised for AMD by HESW, resulting in the highest DERs observed in our experiments. Although RT4 and J82 were similarly susceptible to AMD, they were not sensitised to this drug by shock waves. In contrast, we found considerable DERs of IFN-α for RT4 and J82, but not for MGH-U1. In addition, HESW enhanced the inhibitory effect of TNF-α on the proliferation of RT4 cells.

After shock wave exposure, regeneration processes start to repair cellular damage (Holmes *et al.*, 1992). For this reason, we evaluated the effect of drug addition at various times after HESW treatment for selected combinations of drugs and cell lines. The resulting DERs are displayed in Figure 3. The enhancement ratios gradually diminished during the period

**Figure 2** Dose–response curves for J82 and CDDP. (○) refer to cells without HESW exposure; (□) refer to HESW-treated cells (200 pulses, 0.6 mJ $min^{-2}$). Cells without CDDP incubation served as reference for proliferation (= 1.0).

**Table I** CDDP sensitivity of cell lines pretreated with various shock wave application modes: 0, control; 1, 200 pulses with 0.3 mJ $mm^{-2}$; 2, 1,000 pulses with 0.3 mJ $mm^{-2}$; 3, 200 pulses with 0.6 mJ $mm^{-2}$. After seeding in 96-well plates and attachment the drug was added at a series of dilutons; after a further 72 h incubation cell proliferation was evaluated by the MTT test and the inhibitory concentrations were determined

| *CDDP* | *RT4* | | | *J82* | | | *MGH-U1* | | |
|---|---|---|---|---|---|---|---|---|---|
| *(n = 5)* | *μM* | *σ* | *DER* | *μM* | *σ* | *DER* | *μM* | *σ* | *DER* |
| $IC_{20}$ | | | | | | | | | |
| 0 | 0.48 | *0.06* | | 0.23 | *0.03* | | 0.87 | *0.08* | |
| 1 | 0.38 | *0.07* | (1.3) | 0.19 | *0.03* | – | 0.54 | *0.14* | 1.6 |
| 2 | 0.24 | *0.05* | 2.0 | 0.12 | *0.02* | 1.9 | 0.57 | *0.25* | – |
| 3 | 0.28 | *0.04* | 1.7 | 0.12 | *0.03* | 1.9 | 0.45 | *0.26* | (1.9) |
| $IC_{50}$ | | | | | | | | | |
| 0 | 1.71 | *0.14* | | 0.77 | *0.08* | | 2.66 | *0.18* | |
| 1 | 1.52 | *0.13* | – | 0.70 | *0.13* | – | 2.10 | *0.23* | (1.3) |
| 2 | 1.20 | *0.20* | 1.4 | 0.53 | *0.09* | 1.5 | 1.83 | *0.40* | 1.5 |
| 3 | 1.23 | *0.19* | 1.4 | 0.50 | *0.11* | 1.5 | 1.84 | *0.31* | 1.4 |
| $IC_{80}$ | | | | | | | | | |
| 0 | 5.76 | *0.97* | | 2.58 | *0.35* | | 10.6 | *1.1* | |
| 1 | 6.00 | *1.02* | – | 2.60 | *0.43* | – | 10.5 | *1.4* | – |
| 2 | 5.81 | *0.84* | – | 2.15 | *0.49* | – | 8.9 | *2.4* | – |
| 3 | 5.15 | *0.78* | – | 2.07 | *0.45* | – | 7.5 | *2.5* | (1.4) |

n.d. = not determined. Dose enhancement ratios were calculated from the inhibitory concentrations. Figures indicate a significant difference at $P < 0.01$; figures in parentheses indicate a significant difference at $P < 0.05$; –, no significant difference between shock wave-treated and control cells.

**Table II** MMC sensitivity of cell lines; see Table I for explanation

| *CDDP* | *RT4* | | | *J82* | | | *MGH-U1* | | |
|---|---|---|---|---|---|---|---|---|---|
| *(n = 5)* | *μM* | *σ* | *DER* | *μM* | *σ* | *DER* | *μM* | *σ* | *DER* |
| $IC_{20}$ | | | | | | | | | |
| 0 | 0.15 | *0.02* | | 0.096 | *0.011* | | 0.26 | *0.04* | |
| 1 | 0.14 | *0.04* | – | 0.083 | *0.009* | – | 0.20 | *0.07* | – |
| 2 | 0.10 | *0.02* | 1.5 | 0.051 | *0.007* | 1.9 | 0.15 | *0.03* | 1.7 |
| 3 | 0.11 | *0.04* | – | 0.067 | *0.012* | 1.4 | 0.19 | *0.06* | – |
| $IC_{50}$ | | | | | | | | | |
| 0 | 0.54 | *0.05* | | 0.36 | *0.054* | | 0.79 | *0.06* | |
| 1 | 0.52 | *0.10* | – | 0.33 | *0.066* | – | 0.52 | *0.16* | (1.5) |
| 2 | 0.42 | *0.05* | 1.3 | 0.22 | *0.071* | 1.6 | 0.41 | *0.12* | 1.9 |
| 3 | 0.43 | *0.09* | – | 0.29 | *0.077* | – | 0.53 | *0.13* | (1.5) |
| $IC_{80}$ | | | | | | | | | |
| 0 | 1.83 | *0.23* | | 1.47 | *0.13* | | 4.2 | *0.50* | |
| 1 | 1.90 | *0.42* | – | 1.36 | *0.24* | – | 3.5 | *0.69* | – |
| 2 | 1.63 | *0.24* | – | 0.96 | *0.20* | 1.5 | 3.4 | *0.55* | (1.2) |
| 3 | 1.69 | *0.35* | – | 1.25 | *0.25* | – | 3.8 | *1.0* | – |

**Table III** AMD sensitivity of cell lines; see Table I for explanation

| *CDDP* | *RT4* | | | *J82* | | | *MGH-U1* | | |
|---|---|---|---|---|---|---|---|---|---|
| *(n = 5)* | *μM* | *σ* | *DER* | *μM* | *σ* | *DER* | *μM* | *σ* | *DER* |
| $IC_{20}$ | | | | | | | | | |
| 0 | 7.4 | *0.8* | | 1.7 | *0.22* | | 4.8 | *0.42* | |
| 1 | 7.2 | *0.7* | – | 1.4 | *0.40* | – | 1.2 | *0.17* | 4.0 |
| 2 | 5.5 | *1.4* | (1.3) | 1.4 | *0.39* | – | 0.62 | *0.26* | 7.7 |
| 3 | 5.7 | *1.8* | – | 1.3 | *0.20* | (1.3) | 0.50 | *0.31* | 9.6 |
| $IC_{50}$ | | | | | | | | | |
| 0 | 22.3 | *2.5* | | 8.8 | *1.3* | | 11.5 | *0.77* | |
| 1 | 23.0 | *2.1* | – | 7.5 | *2.2* | – | 5.2 | *0.30* | 2.2 |
| 2 | 19.5 | *4.0* | – | 6.9 | *1.8* | – | 3.0 | *0.40* | 3.8 |
| 3 | 20.7 | *4.7* | – | 7.2 | *1.4* | – | 3.1 | *0.48* | 3.7 |
| $IC_{80}$ | | | | | | | | | |
| 0 | 65.4 | *7.2* | | 49 | *9.5* | | 27.1 | *2.4* | |
| 1 | 71.4 | *8.2* | – | 40 | *8.5* | – | 16.0 | *1.6* | 1.7 |
| 2 | 66.2 | *12.0* | – | 35 | *9.6* | – | 11.9 | *1.8* | 2.3 |
| 3 | 70.5 | *13.8* | – | 55 | *21.0* | – | 10.8 | *1.9* | 2.5 |

**Table IV** IFN-α sensitivity of cell lines; see Table I for explanation

| *IFN-α* (n = *4*) | RT4 *U ml⁻¹* | *σ* | *DER* | J82 *U ml⁻¹* | *σ* | *DER* | MGH-U1 *U ml⁻¹* | *σ* | *DER* |
|---|---|---|---|---|---|---|---|---|---|
| $IC_{20}$ | | | | | | | | | |
| 0 | 160 | *20* | | 700 | *100* | | $1.5 \times 10^4$ | *$2.4 \times 10^3$* | |
| 1 | 98 | *20* | 1.6 | 720 | *110* | – | $1.6 \times 10^4$ | *$3.2 \times 10^3$* | – |
| 2 | 59 | *8.5* | 2.7 | 370 | *95* | 1.9 | $2.1 \times 10^4$ | *$4.6 \times 10^3$* | – |
| 3 | 55 | *14* | 2.9 | 370 | *32* | 1.9 | $1.4 \times 10^4$ | *$2.5 \times 10^3$* | – |
| $IC_{50}$ | | | | | | | | | |
| 0 | 1,300 | *140* | | 8,200 | *680* | | $8.0 \times 10^4$ | *$1.0 \times 10^4$* | |
| 1 | 930 | *120* | (1.4) | 7,600 | *710* | – | $7.8 \times 10^4$ | *$1.2 \times 10^4$* | – |
| 2 | 650 | *74* | 2.0 | 3,700 | *720* | 2.2 | $8.4 \times 10^4$ | *$1.5 \times 10^4$* | – |
| 3 | 880 | *105* | 1.5 | 4,500 | *370* | 1.8 | $8.1 \times 10^4$ | *$8.4 \times 10^3$* | – |
| $IC_{80}$ | | | | | | | | | |
| 0 | $1.4 \times 10^4$ | *960* | | $1.1 \times 10^6$ | *$9.0 \times 10^4$* | | | n.d. | |
| 1 | $1.2 \times 10^4$ | *1400* | – | $9.4 \times 10^5$ | *$9.5 \times 10^4$* | (1.2) | | n.d. | |
| 2 | $1.0 \times 10^4$ | *980* | 1.4 | $7.9 \times 10^5$ | *$1.2 \times 10^5$* | 1.4 | | n.d. | |
| 3 | $1.0 \times 10^4$ | *1200* | 1.4 | $5.8 \times 10^5$ | *$7.2 \times 10^4$* | 1.9 | | n.d. | |

**Table V** TNF-α sensitivity of RT4 cells; see Table I for explanation

| *TNF-α* (n = *3*) | RT4 *U ml⁻¹* | *σ* | *DER* |
|---|---|---|---|
| $IC_{20}$ | | | |
| 0 | 1,050 | *180* | |
| 1 | 770 | *110* | – |
| 2 | 420 | *110* | (2.5) |
| 3 | 390 | *80* | (2.7) |
| $IC_{50}$ | | | |
| 0 | $4.9 \times 10^3$ | *570* | |
| 1 | $4.4 \times 10^3$ | *550* | – |
| 2 | $2.4 \times 10^3$ | *780* | (2.0) |
| 3 | $2.7 \times 10^3$ | *490* | (1.8) |
| $IC_{80}$ | | | |
| 0 | n.d. | | |
| 1 | n.d. | | |
| 2 | n.d. | | |
| 3 | n.d. | | |

of 40 h tested in this study. The decline was very rapid for J82 and CDDP and for MGH-U1 and AMD. The loss of increased IFN-α sensitivity of RT4 cells extended over a longer period of time.

To assess the effects of concomitant treatments and treatments in direct sequence, the HESW exposure was performed with the suspension system. In this way, adequate drug delivery to the cells and short handling times could be guaranteed. The modulation of cell proliferation by different treatment regimens is depicted in Figure 4. Single treatments of either drug or shock waves produced, if any, only slight growth inhibition at the chosen concentrations and HESW parameters. For all combinations of cell lines and drugs, the inhibitory effect was smallest when drug incubation preceded HESW exposure, and became most pronounced for simultaneous treatments. The growth reduction caused by concomitant CDDP and HESW treatment was diminished by subsequent HESW exposure after drug removal (significant for J82: $P<0.05$). Results that indicate synergistic interactions of the two treatment modalities are marked in Figure 4.

**Figure 3** Dose enhancement ratio dependent on different times of drug addition (Δ, J82 and CDDP; □, MGH-U1 and AMD; O, RT4 AND IFN) after HESW exposure; 0 h denotes seeding of cells directly into drug-containing medium; other time points refer to drug addition after cell attachment.

**Figure 4** Relative proliferation after single or combined treatment regimens and a further 72 h incubation as determined by the MTT test. Control cells receiving no treatment served as a reference (= 1.0). Synergistic effects under assumption of the additivity model are marked with asterisks. $^*P<0.05$, $^{**}P<0.01$.

The results of flow cytometric measurements are summarised in Table VI. The extent of cell damage, characterised as the percentage of PI-positive cells and the intensity of fluorescein fluorescence, was not altered by a 12 min incubation with CDDP. Shock wave exposure, however, affected these parameters. The portion of cells accumulating PI was moderately higher for the suspension system than for the pellet system and for RT4 cell as compared with J82 cells. Cells exposed in suspension showed a slightly decreased fluorescein fluorescence. Contemporaneous CDDP incubation produced a minor increase in PI-stained cells.

### Discussion

Previous *in vitro* studies have demonstrated that HESW can cause various types of cell damage. Apart from immediately lethal effects such as fragmentation of cells, permeabilisation of plasma membrane, swollen mitochondria with distorted cristae, disturbed mitochondrial membrane potential, altered vimentin structure, cytoplasmic cisternae and nuclear changes have been described (Russo *et al.*, 1987; Randazzo *et al.*, 1988; Bräuner *et al.*, 1989; Kohri *et al.*, 1990; Yu *et al.*, 1991; Steinbach *et al.*, 1992). Moreover, metabolic changes in HESW-treated tumours have been reported (Smits *et al.*, 1991). However, first experiments with combinations of HESW and cytostatic drugs did not yield uniform results. For example, in experimental systems enhanced effects of CDDP or adriamycin in combination with HESW were reported while in others no such effects could be observed (Randazzo *et al.*, 1988; Wilmer *et al.*, 1989; Berens *et al.*, 1989; Holmes *et al.*, 1990; Lee *et al.*, 1990; Chung *et al.*, 1991; Gambihler & Delius, 1992). Additional data are needed to achieve a thorough understanding of the interactions of HESW and tumour cells. In this study employing a series of bladder cancer cell lines, the effects of certain drugs in combination with HESW exposure seemed to be similar for all cell lines; however, the effects of others were highly dependent on the specific cell line under investigation.

The sensitivity to CDDP could be uniformly increased by HESW pretreatment for all cell lines. This sensitising effect diminished rapidly during the first hours after HESW exposure. In addition, experiments with high CDDP concentrations over a short incubation time revealed that the combined growth-inhibitory effect becomes most pronounced for simultaneous treatments. HESW exposure prior to CDDP incubation produced a modest growth inhibition, whereas CDDP treatment before HESW exposure did not result in reduced proliferation significantly different from those of either treatment modality alone. This observation is in accordance with the results of Lee *et al.* (1990) and strongly supports the assumptions of Gambihler & Delius (1992) that attribute enhanced CDDP toxicity in combination with HESW to temporarily increased plasma membrane permeability. CDDP is a relatively polar, i.e. hydrophilic, molecule that normally crosses the lipid bilayer of the plasma membrane at a slow rate, mainly by passive diffusion (Hecquet *et al.*, 1986). HESW exposure could transiently permeabilise the cell membrane, thus reducing the diffusion barrier for CDDP and increasing CDDP influx in analogy with results obtained by ultrasound exposure (Ellwart *et al.*, 1987; Kober *et al.*, 1989; Fahnestock *et al.*, 1989) or electropermeabilisation of cells (Melvik *et al.*, 1986). This interpretation seems reasonable regarding the enhanced proliferation capacity following a second HESW administration after concomitant CDDP/HESW treatment. As intracellular CDDP shows only slow efflux rates (Troger *et al.*, 1992), a second shock wave permeabilisation of the cell membrane after removal of extracellular drug could increase efflux of active CDDP before being bound to intracellular target sites and consequently increase the survival rate of the cells. Recently, analogous observations were made for electropermeabilisation of cells (Melvik *et al.*, 1992). Flow cytometric measurements supply an additional clue to membrane permeabilisation by HESW. PI influx is clearly increased after HESW treatment, as well as fluorescein efflux, though to a lesser extent. The RT4 cell line showed higher PI uptake, reflecting its higher susceptibility to concomitant and subsequent HESW/CDDP treatments. As previously reported (Gambihler *et al.*, 1992), there is a fraction of fluorescein-positive cells that is also PI positive but obviously proliferates, indicating that these cells are able to recover from the membrane damage that leads to PI uptake. Again, there is a remarkable similarity to electropermeabilisation, the effect of which is entirely reversible (Melvik *et al.*, 1986). However, this sublethal membrane damage could lead to the introduction of a potentially lethal dose of CDDP into the cell.

The fact that there remains an enhanced sensitivity to CDDP even several hours after HESW treatment, as well as the combined effects of HESW and the other drugs, may be attributed to other shock wave-related effects. MMC exhibits a more lipophilic character than CDDP and accumulates more readily in cells (Wallner *et al.*, 1987). MMC showed a moderately higher efficiency similar for all cell lines after HESW pretreatment. Concurrent HESW and high-dose MMC treatment could not result in synergistic effects similar to those of CDDP (data not shown). Therefore, we have to assume that there occurs a cellular alteration that may be similar to those observed in hyperthermic or radiation enhancement of MMC action (Barlogie *et al.*, 1980). Besides changes in drug accumulation, increased drug activation, inhibition of repair processes and improved accessibility of DNA targets are discussed (Lokich & Byfield, 1991).

AMD, in contrast, is a highly lipophilic substance that diffuses rapidly through the plasma membrane (Orlowski *et al.*, 1988). Sensitivity to AMD could be enhanced only in MGH-U1, reaching the highest DER values observed in this study. The DER of AMD decreases very rapidly during the first hours after HESW exposure. High-dosage combination treatments resulted in synergistic growth-inhibitory effects similar to those reported for CDDP treatments. However, even AMD pretreatment produced a synergistic effect, and a

**Table VI** Evaluation of flow cytometry measurements, percentage of PI-stained cells and mean relative intensity of fluorescein fluorescence for different treatments

| | *J82* | | | | *RT4* | | | |
|---|---|---|---|---|---|---|---|---|
| | *PI positive* | | *Fluorescein intensity* | | *PI positive* | | *Fluorescein intensity* | |
| | *(%)* | *σ* | $I_{tr.}/I_{contr.}$ | *σ* | *(%)* | *σ* | $I_{tr.}/I_{contr.}$ | *σ* |
| Control | 3.9 | *1.7* | – | | 4.6 | *0.4* | – | |
| CDDP | 4.3 | *2.2* | 1.03 | *0.06* | 4.6 | *1.2* | 1.13 | *0.06* |
| HESW, pellet | 10.8 | *6.0* | 0.96 | *0.08* | 18.4 | *7.5* | 1.0 | *0.04* |
| HESW, suspension | 14.8 | *5.3* | 0.89 | *0.03* | 26.0 | *4.2* | 0.85 | *0.10* |
| HESW + CDDP | 17.4 | *4.4* | 0.90 | *0.12* | 29.1 | *6.0* | 0.90 | *0.03* |

'CDDP', 12 min incubation with 15 μM CDDP in suspension; HESW, pellet/suspension, cell pellet/suspension exposed to 200 shock wave pulses of 0.6 mJ $min^{-2}$; HESW + CDDP, cell suspension after simultaneous HESW and CDDP treatment.

second HESW exposure after simultaneous AMD/HESW treatment further enhanced growth inhibition, thus indicating an additional damage to the cells. Electropermeabilisation does not result in increased AMD sensitivity of cells (Orlowski et al., 1988). As a consequence, membrane damage can be excluded as the main component mediating the combined action of HESW and AMD on MGH-U1 cells. Concerning combined treatments with hyperthermia or radiation and AMD, a reduction in the ability to absorb sublethal damage is discussed by Ziegler et al. (1987) and Komatsu et al. (1988). This seems reasonable since AMD binds preferentially to nucleolar chromatin and impairs ribosomal RNA synthesis (Yu & Bender, 1990). However, a reduced capacity to repair damage caused by HESW should apply to all cell lines, in accordance with results obtained for combination therapies involving radiation or heat. On the other hand, AMD shows a range of highly specific cellular reactions, such as enzymatic reduction to a free radical species (Flitter & Mason, 1988) and translocation of protein B23 (Yung et al., 1990). Perhaps 'side effects' of this kind may play a crucial role in the reaction of MGH-U1 to HESW and AMD treatment. Therefore, as a hypothesis, we have to presume an inherent characteristic of the cell line MGH-U1 to be responsible for the observed effects in combination with HESW.

The mechanisms of action of both IFN-α and TNF-α, in particular their cytostatic and cytotoxic effects, are not yet fully understood (Baron et al., Fiers, 1991; Schütze et al., 1992; Weil, 1992). Various intracellular reactions are triggered after binding to specific transmembrane receptors. Inhibition or induction of a range of proteins, enzymes and oncogenes, as well as changes in the cytoskeleton, have been described. It remains to be elucidated why some cell lines are sensitive to certain cytokines while others are not. We observed that growth-inhibitory effects on those cell lines that were susceptible to a cytokine could be further enhanced by HESW pretreatment. On the other hand, shock waves could not make insensitive cell lines sensitive to either IFN-α or TNF-α. The enhanced sensitivity to IFN-α diminishes less rapidly than caused by CDDP and AMD. This is a first indication that shock waves interfere with the pathways underlying the cytotoxicity of cytokines than chemotherapeutic agents. Shock waves may induce an additional disturbance of protein synthesis or enzymatic activity, thus contributing to the cytolytic effect of cytokines. Nevertheless, in recent research, there is some evidence that the plasma membrane may also contribute to the cytotoxicity of both IFN-α and TNF-α (Taylor-Papadimitriou & Rozengurt, 1985; Anghileri & Robert, 1987; Stalc et al., 1992). A shock wave-induced change in this target may concur with the theory that the action of cytokines is to bring about an additional effect. In a model system of experimental tumours, complete remission could be achieved by application of shock waves after injection of TNF-α (Oosterhof et al., 1991). This effect is ascribed to severe damage to tumour vascularisation as a consequence of combined HESW and TNF-α action (Steinbach et al., 1993). Though this is probably the decisive factor, we could show that direct cytotoxic effects of cytokines can also be augmented by HESW.

The data in Tables I–V suggest that there is a tendency for DER values to decrease with increasing cytotoxicity. A possible explanation is the spatial distribution of the energy density of the shock wave and its relation to cellular damage. Cells exposed as a pellet have a relatively fixed position within the focal region of the lithotripter. With respect to the rapid decline in the energy density when moving away from the acoustic axis of the propagating shock wave (see Figure 1) the whole cell population is not damaged to the same extent by HESW exposure. Thus, an HESW-treated specimen is expected to show a possibly higher but less homogeneous sensitivity to subsequent drug exposure than cells without pretreatment. This observation might indicate the need for a multifocal exposure of tumours in *in vivo* systems in order to achieve a uniform reaction of the tissue and to establish a reproducible treatment protocol.

Previous studies and our results suggest that cell membrane permeabilisation is the most prominent alteration induced by essentially sublethal doses of HESW. Cells recover from this type of damage rapidly. Thus, concurrent treatment regimens with HESW and hydrophilic drugs look promising since HESW can regionally render tissue more susceptible to the drug with the prospect of reduced systemic toxicity. Besides, there seem to be various intracellular HESW-mediated alterations that interact with cytotoxic drugs. Addition of several types of sublethal damage and impaired repair mechanisms may explain the effects of such combined treatments. Further studies are necessary to achieve a profound understanding of how shock waves interact with cells and cytostatic agents. Moreover, the physical parameters of shock waves have to be taken into consideration in order to optimise the protocol for a shock wave-assisted chemotherapy.

This work was supported by Siemens Medizintechnik, Erlangen, Germany.

### References

ANGHILERI, L.J. & ROBERT, J. (1987). Effects of tumor necrosis factor on tumor cell plasma membrane permeability. *Tumori*, **73**, 269–271.

BARLOGIE, B., CORRY, P.M. & DREWINKO, B. (1980). *In vitro* thermochemotherapy of human colon cancer cells with cis-dichlorodiammineplatinum(II) and mitomycin C. *Cancer Res.*, **10**, 1165–1168.

BARON, S., TYRING, S.K., FLEISCHMANN, W.R., COPPENHAVER, D.H., NIESEL, D.W., KLIMPEL, G.R., STANTON, G.J. & HUGHES, T.K. (1991). The interferons, mechanisms of action and clinical applications. *JAMA*, **266**, 1375–1383.

BERENS, M.E., WELANDER, C.E., GRIFFIN, A.S. & MCCULLOUGH, D.L. (1989). Effect of acoustic shock waves on clonogenic growth and drug sensitivity of human tumor cells *in vitro*. *J. Urol.*, **142**, 1090–1094.

BRÄUNER, T., BRÜMMER, F. & HÜLSER, D.F. (1989). Histopathology of shock wave treated tumor cell suspensions and multicell tumor spheroids. *Ultrasound Med. Biol.*, **15**, 451–460.

BRÜMMER, F., BRÄUNER, T. & HÜLSER, D.F. (1990). Biological effects of shock waves. *World J. Urol.*, **8**, 224–232.

CHUNG, W.S., KIM, H.I., KIM, S.J., KIM, N.S., LEE, M.S. & KIM, S.J. (1991). Effects of high energy shock waves on *in vitro* tumor cell chemosensitivity and *in vivo* tumor growth. *J. Korean Med. Assoc.*, **34**, 190–196.

ELLWART, J.W., BRETTEL, H. & KOBER, L.O. (1987). Cell membrane damage by ultrasound at different cell concentrations. *Ultrasound Med. Biol.*, **14**, 43–50.

FAHNESTOCK, M., RIMER, V.G., YAMAWAKI, R.M., ROSS, P. & EDMONDS, P.D. (1989). Effects of ultrasound exposure *in vitro* on neuroblastoma cell membranes. *Ultrasound Med. Biol.*, **15**, 133–144.

FIERS, W. (1991). Tumor necrosis factor, characterization at the molecular, cellualr and *in vivo* level. *FEBS Lett.*, **285**, 199–212.

FLANIGAN, R.C., PAVLIK, E.J., VAN NAGELL, J.R., KEATON, K. & KENADY, D.E. (1986). Proliferation, esterase activity, and propidium iodide exclusion in urologic tumor cells after *in vitro* exposure to chemotherapeutic agents. *J. Urol.*, **135**, 1091–1100.

FLITTER, W.D. & MASON, R.P. (1988). The enzymatic reduction of actinomycin D to a free radical species. *Arch. Biochem. Biophys.*, **267**, 632–639.

FOLBERTH, W., KÖHLER, G., ROHWEDDER, A. & MATURA, E. (1992). Pressure distribution and energy flow in the focal region of two different electromagnetic shock wave sources. *J. Stone Dis.*, **4**, 1–7.

GAMBIHLER, S. & DELIUS, M. (1992). *In vitro* interaction of lithotripter shock waves and cytotoxic drugs. *Br. J. Cancer*, **66**, 69–73.

GAMBIHLER, S., DELIUS, M. & ELLWART, J.W. (1992). Transient increase in membrane permeability of L1210 cells upon exposure to lithotripter shock waves *in vitro*. *Naturwissenschaften*, **79**, 328-329.

HECQUET, B., LEROY, A., LEFEBVRE, J.L., PEYRAT, J.P. & ADENIS, L. (1986). Uptake of platinum compounds in human tumors, *in vitro* study. *Bull. Cancer*, **73**, 535-541.

HOLMES, R.P., YEAMAN, L.I., LI, W.J., HART, L.J., WALLEN, C.A., WOODRUFF, R.D. & MCCULLOUGH, D.L. (1990). The combined effects of shock waves and cisplatin therapy on rat prostate tumors. *J. Urol.*, **144**, 159-163.

HOLMES, R.P., YEAMAN, L.D., TAYLOR, R.G. & MCCULLOUGH, D.L. (1992). Altered neutrophil permeability following shock wave exposure *in vitro*. *J. Urol.*, **147**, 733-737.

HOSHI, S., ORIKASA, S., KUWAHARA, M., SUZUKI, K., SHIRAI, S., YOSHIKAWA, K. & NOSE, M. (1992). Shock wave and THP-adriamycin for treatment of rabbit's bladder cancer. *Jpn. J. Cancer Res.*, **83**, 248-250.

IRO, H., SCHNEIDER, H.T., FODRA, C., WAITZ, G., NITSCHE, N., HEINRITZ, H.H., BENNINGER, J. & ELL, C. (1992). Shockwave lithotripsy of salivary duct stones. *Lancet*, **339**, 1333-1336.

KOBER, L.O., ELLWART, J.W. & BRETTEL, H. (1989). Effects of the pulse length of ultrasound on cell membrane damage *in vitro*. *J. Acoust. Soc. Am.*, **86**, 6-7.

KOHRI, K., UEMURA, T., IGUCHI, M. & KURITA, T. (1990). Effect of high energy shock waves on tumor cells. *Urol. Res.*, **18**, 101-105.

KOMATSU, K., MILLER, R.C. & HALL, E.J. (1988). The oncogenic potential of a combination of hyperthermia and chemotherapeutic agents. *Br. J. Cancer*, **57**, 59-63.

LEE, K.E., SMITH, P. & COCKETT, A.T.K. (1990). Influence of high energy shock waves and cisplatin on antitumor effect in murine bladder cancer. *Urology*, **36**, 440-444.

LIN, C.W., LIN, J.C. & PROUT, G.R. (1985). Establishment and characterization of four human bladder tumor cell lines and sublines with different degrees of malignancy. *Cancer Res.*, **45**, 5070-5079.

LINGEMAN, J.E., MCATEER, J.A., KEMPSON, S.A. & EVAN, A.P. (1988). Bioeffects of extracorporeal shock wave lithotripsy. *Urol. Clin. North Am.*, **15**, 507-514.

LOKICH, J.J. & BYFIELD, J.E. (eds) (1991). *Combined Modality Cancer Therapy*. Precept Press: Chicago.

MELVIK, J.E., PETTERSEN, E.O., GORDON, P.B. & SEGLEN, P.O. (1986). Increase in cis-dichlorodiammineplatinum(II) cytotoxicity upon reversible electropermeabilization of the plasma membrane in cultured human NHIK 3025 cells. *Eur. J. Cancer Clin. Oncol.*, **22**, 1523-1530.

MELVIK, J.E., DORNISH, J.M. & PETTERSEN, E.O. (1992). The binding of cis-dichlorodiammineplatinum(II) to extracellular and intracellular components in relation to drug uptake and cytotoxicity *in vitro*. *Br. J. Cancer*, **66**, 260-265.

MOSMANN, T. (1983). Rapid colorimetric assay for cellular growth and survival: application to proliferation and cytotoxicity assays. *J. Immunol. Methods*, **65**, 55-63.

O'TOOLE, C., PRICE, Z.H., OHNUKI, Y. & UNSGAARD, B. (1978). Ultrastructure, karyology and immunology of a cell line originated from a human transitional cell carcinoma. *Br. J. Cancer*, **38**, 64-76.

OOSTERHOF, G.O., SMITS, G.A., DE RUYTER, A.E., SCHALKEN, J.A. & DEBRUYNE, F.M. (1991). Effects of high energy shock waves combined with biological response modifiers in different human kidney cancer xenografts. *Ultrasound Med. Biol.*, **17**, 391-399.

ORLOWSKI, S., BELEHRADEK, J., PAOLETTI, C. & MIR, L.M. (1988). Transient electropermeabilization of cells in culture, increase of the cytotoxicity of anticancer drugs. *Biochem. Pharmacol.*, **37**, 4727-4733.

RANDAZZO, R.F., CHAUSSY, C.G., FUCHS, G.J., BHUTA, S.M., LOVREKOVICH, H. & DEKERNION, J.B. (1988). The *in vitro* and *in vivo* effects of extracorporeal shock waves on malignant cells. *Urol. Res.*, **16**, 419-426.

RIGBY, C.C. & FRANKS, L.M. (1970). A human tissue culture cell line from a transitional cell tumour of the urinary bladder: growth, chromosome pattern and ultrastructure. *Br. J. Cancer*, **24**, 746-754.

RUSSO, P., HESTON, W.D.W. & FAIR, W.R. (1985). Suppression of *in vitro* and *in vivo* tumor growth by high energy shock waves. *Surg. Forum*, **36**, 646-648.

RUSSO, P., MIES, C., HURYK, R., HESTON, W.D.W. & FAIR, W.R. (1987). Histopathologic and ultrastructural correlates of tumor growth suppression by high energy shock waves. *J. Urol.*, **137**, 338-341.

SACKMAN, M. & PAUMGARTNER, G. (1992). Biliary lithotripsy by extracorporeal generated shock waves. *Recenti Prog. Med.*, **83**, 400-406.

SCHÜTZE, S., MACHLEIDT, T. & KRÖNKE, M. (1992). Mechanisms of tumor necrosis factor action. *Semin. Oncol.*, **19** (suppl. 4), 16-24.

SIMON, J., CORBUSIER, A., MERDES-LEAL, A., VAN DEN BOSSCHE, M., WESPES, E., VAN REGEMORTER, G. & SCHULMAN, C.C. (1989). Extracorporeal shock wave lithotripsy for urinary stone disease. *Eur. Urol.*, **16**, 7-11.

SMITS, G.A., HEERSCHAP, A., OOSTERHOF, G.O., RUYS, J.H., HILBERS, C.W., DEBRUYNE, F.M. & SCHALKEN, J.A. (1991). Early metabolic response to high energy shock waves in a human kidney xenograft monitored by $^{31}P$ magnetic resonance spectroscopy. *Ultrasound Med. Biol.*, **17**, 791-801.

STALC, A., SENTJURC, M., SERSA, G. & NOVAKOVIC, S. (1992). The influence of TNF on the membrane fluidity of tumor cells. *Cancer Lett.*, **65**, 183-187.

STEINBACH, P., HOFSTÄDTER, F., NICOLAI, H., RÖSSLER, W. & WIELAND, W. (1992). *In vitro* investigations on cellular damage induced by high energy shock waves. *Ultrasound Med. Biol.*, **18**, 691-699.

STEINBACH, P., HOFSTÄDTER, F., NICOLAI, H., RÖSSLER, W. & WIELAND, W. (1993). Determination of the energy dependent extent of vascular damage caused by high energy shock waves in an umbilical cord model. *Urol. Res.*, **21**, 279-282.

TAYLOR-PAPADIMITRIOU, J. & ROZENGURT, E. (1985). Interferons as regulators of cell growth and differentiation. In *Interferons: Their Impact in Biology and Medicine*. Taylor-Papadimitriou, J. (ed.) pp. 81-98. Oxford University Press: Oxford.

TROGER, V., FISCHEL, J.L., FORMENTO, P., GIOANNI, J. & MILANO, G. (1992). Effects of prolonged exposure to cisplatin on cytotoxicity and intracellular drug concentration. *Eur. J. Cancer*, **28**, 82-86.

WALLNER, K.E., BANDA, M. & LI, G.C. (1987). Hyperthermic enhancement of cell killing by mitomycin C in mitomycin C-resistant Chinese hamster ovary cells. *Cancer Res.*, **47**, 1308-1312.

WARLTERS, A., MORRIS, D.L., CAMERON-STRANGE, A. & LYNCH, W. (1992). Effect of electrohydraulic and extracorporeal shock waves on gastrointestinal cancer cells and their response to cytotoxic agents. *Gut*, **33**, 791-793.

WEIL, D. (1992). What's new about tumor necrosis factor? *Eur. Cytokine Netw.*, **3**, 347-351.

WELANDER, C.E., MORGAN, T.M., HOMESLEY, H.D., TROTTA, P.P. & SPIEGEL, R.J. (1985). Combined recombinant human interferon $alpha_2$ and cytotoxic agents studied in a clonogenic assay. *Int. J. Cancer*, **35**, 721-729.

WILMER, A., GAMBIHLER, S., DELIUS, M. & BRENDEL, W. (1989). *In vitro* cytotoxic activity of lithotripter shock waves combined with adriamycin or with cisplatin on L1210 mouse leukemia cells. *J. Cancer Res. Clin. Oncol.*, **115**, 229-234.

YU, D.S., CHEN, A., SU, C.J., CHANG, S.Y., MA, C.P. & CHU, T.M. (1991). Effects of high energy shock waves on murine renal cell carcinoma. *Urology*, **38**, 571-576.

YU, F.L. & BENDER, W. (1990). Actinomycin D binding *in vitro*: active chromatin preferred. *Biochem. Int.*, **20**, 807-815.

YUNG, B.Y.M., BOR, A.M.S. & CHAN, P.K. (1990). Short exposure to actinomycin D induces 'reversible' translocation of protein B23 as well as 'reversible' inhibition of cell growth and RNA synthesis in HeLa cells. *Cancer Res.*, **50**, 5987-5991.

ZIEGLER, W., BIRKENFELD, P. & TROTT, K.R. (1987). The effect of combined treatment of HeLa cells with actinomycin D and radiation upon survival and recovery from radiation damage. *Radiother. Oncol.*, **10**, 141-148.